# EARL compliance on the Biograph Vision Quadra PET-CT: phantom study for static and continuous bed motion acquisitions

**DOI:** 10.3389/fnume.2025.1646628

**Published:** 2025-07-29

**Authors:** Beverley F. Holman, Tamar Willson, Bruno Ferreira, Neil Davis, Hemangini Natarajan, Jannat Khan, Thomas Wagner, Daniel McCool

**Affiliations:** ^1^ Nuclear Medicine, Royal Free Hospital, London, United Kingdom; ^2^ School of Medicine, UCL, London, United Kingdom; ^3^ Radiation Physics & Radiobiology, Imperial College Healthcare NHS Trust, London, United Kingdom; ^4^ Centre for Medical Imaging, UCL, London, United Kingdom

**Keywords:** PET-CT, LAFOV, Quadra, EARL, harmonisation, image quality

## Abstract

**Purpose:**

Long axial field-of-view (LAFOV) PET systems like the Siemens Biograph Vision Quadra offer unprecedented sensitivity and imaging capabilities, but compliance with EARL standards across all acquisition modes remains unexplored. This study aimed to identify reconstruction parameters meeting EARL 1 and 2 compliance for static and continuous bed motion (CBM) acquisitions in High Sensitivity (HS) and Ultra-High Sensitivity (UHS) modes on the Quadra. The research focused on optimising image quality while maintaining compliance with quantitative standards.

**Methods:**

The International Electrotechnical Commission (IEC) body phantom was filled with ^18^F-FDG in a 10:1 sphere-to-background activity ratio and scanned at five positions across the field of view (FOV) using static and CBM acquisitions in HS and UHS modes. Reconstructions used standard clinical parameters, varied with Gaussian filters (1–7 mm) and matrix sizes (440, 220, 128). EARL compliance was assessed with the EARL tool to evaluate SUV recovery coefficients (RCSUVmean, RCSUVmax, RCSUVpeak). Patient images were reconstructed using standard and EARL-compliant parameters for comparison.

**Results:**

Reconstruction parameters achieving EARL compliance were identified for all acquisition modes, with no differences between static and CBM reconstructions. Achieving EARL compliance required significant image quality reductions, especially for EARL 1, with greater degradation in UHS mode. Patient images reconstructed with EARL-compliant parameters appeared smoother and had reduced contrast compared to clinical reconstructions.

**Conclusion:**

While EARL compliance ensures quantitative standardisation, it significantly reduces image quality, especially on advanced LAFOV PET systems. An updated “EARL 3” standard is needed to reflect the capabilities of modern systems.

## 1 Introduction

The European Association of Nuclear Medicine (EANM) Research Ltd. (EARL) accreditation program provides standardised performance criteria for positron emission tomography (PET) imaging, enabling consistency and comparability across centres and platforms (1). Compliance with EARL criteria is essential for ensuring reliable quantitative imaging, especially in multi-centre clinical trials and longitudinal studies (2).

The first-generation EARL standard, EARL 1, was designed to address the variability in image quality and quantification among older, non-digital PET-CT systems, focusing on reproducibility and standardisation in multi-centre oncology studies. Advancements in PET-CT technology [namely silicon photomultipliers (SiPMs), advanced reconstruction techniques and improved time-of-flight (ToF) resolution] led to significant improvements in image quality. Unfortunately, these advances could not be fully exploited in practice without producing images that were not compliant with the EARL 1 standard. Consequently EARL 2 was introduced: a second-generation standard designed to accommodate the enhanced capabilities of newer PET-CT scanners. EARL 2 allows for better-spatial resolution imaging and improved contrast recovery while maintaining the foundational goal of harmonised quantification (3, 4).

More recently, a new generation of PET-CT scanners has emerged: the long axial field-of-view (LAFOV) systems. These scanners offer extended axial coverage, enabling enhanced sensitivity, whole-body dynamic imaging, and ultra-low-dose capabilities. Such evolutions have the potential to revolutionise both clinical and research applications by improving image quality, reducing scan times and allowing for high temporal resolution dynamic imaging (5, 6). The Biograph Vision Quadra PET-CT (Siemens Healthineers, Erlangen) is a LAFOV digital PET-CT with an axial field of view (FOV) of 106 cm, ToF capabilities of less than 230 ps and system effective sensitivity exceeding 803 cps/kBq (6). Optimising reconstruction parameters to achieve EARL compliance on these advanced systems remains challenging due to the trade-off between maintaining image quality and adhering to stringent quantitative standards particularly across various acquisition modes and settings (7, 8).

Most existing studies on EARL compliance focus on short axial FOV PET-CT systems (1–3). LAFOV PET has only been available since 2019 (5) with the first Siemens Vision Quadra PET-CT (Quadra) installed in Bern in 2020 (6). Originally, the Quadra could only acquire static acquisitions and reconstruct in high sensitivity (HS) mode. In HS mode, the crystals have an acceptance angle of 19°, equivalent to a mean ring difference of 85 (MRD85) (6–8). Studies on EARL compliance in static and HS mode have been performed by both Bern and Groningen (7, 8) with the latter extending the work to compare to clinical data. However, the Quadra's capabilities now extend beyond static imaging to continuous bed motion (CBM) acquisitions, an approach that offers improved uniformity across the large axial FOV (9). Furthermore, the Ultra-High Sensitivity (UHS) mode, with an acceptance angle of 52° (MRD322), presents an opportunity to further leverage the system's enhanced performance and significantly increased sensitivity (10). To date, no work has been published to comprehensively evaluate EARL compliance under these expanded conditions.

This study aims to systematically evaluate reconstruction parameters for both static and CBM acquisitions on the Quadra using phantom data. The analysis includes both HS and UHS modes, considering both EARL1 and EARL2 accreditation, thus yielding eight distinct reconstruction scenarios. The results of this study are expected to inform optimisation strategies for quantitative PET imaging, contributing to the broader goal of achieving consistent and standardised imaging in nuclear medicine.

## 2 Materials and methods

The study was conducted using the International Electrotechnical Commission (IEC) body phantom filled with ^18^F-FDG at a 10:1 activity ratio of spheres to background, following the standardised protocol described by the European Association of Nuclear Medicine (EANM) Research Ltd. (EARL) (11). The spheres in the phantom were centred and scanned in five positions across the 106 cm axial FOV of the Quadra: at ¼ (26.5 cm), ⅓ (35.3 cm), ½ (53 cm), ⅔ (70.7 cm), and ¾ (79.5 cm) of the total FOV length. These locations are specified by EARL and ensure comprehensive evaluation of the scanner's imaging performance across the entire FOV.

For static acquisitions, the phantom was scanned for a duration of 5 min at each location. Continuous bed motion (CBM) acquisitions were performed with a bed speed of 2.2 mm/s for comparison in Ultra-High Sensitivity (UHS) mode and 3.2 mm/s for comparison in High Sensitivity (HS) mode. These speeds were recommended by Siemens Healthineers to achieve comparable image quality between static and CBM acquisitions under the respective sensitivity modes. The goal was to optimise the CBM acquisition parameters to closely match the static imaging performance in terms of EARL compliance. A CT acquisition (quality reference mAs: 65, CarekV and CareDose on, reference kV 120, pitch 0.8) was obtained before the start of each PET acquisition to allow for CT attenuation correction of the PET data.

Once all images were acquired they were reconstructed using a range of parameters. All images were corrected for attenuation, scatter, decay, normalisation, dead time, and randoms. The baseline reconstruction was performed using the standard clinical protocol: Ordered Subset Expectation Maximisation (OSEM) with 4 iterations and 5 subsets, incorporating point spread function (PSF) correction (marketed as TrueX by Siemens) and time-of-flight (ToF). No post-reconstruction filter was initially applied, and the images were reconstructed onto a 440 × 440 matrix. Subsequently, additional reconstructions were performed by applying Gaussian filters with full-width at half-maximum (FWHM) ranging from 1 mm to 7 mm. Each reconstruction was further evaluated using three different matrix sizes: 440 × 440, 220 × 220, and 128 × 128, resulting in a total of 480 unique reconstructions.

The compliance of each reconstruction was assessed using the EARL analysis tool (EANM_QC_TOOLS_V16112018), which evaluates recovery coefficients for SUVmean, SUVmax, and SUVpeak, The recovery coefficient of SUVmean (RCSUVmean) and RCSUVmax were calculated for both EARL 1 and EARL 2 compliance. For RCSUVmean, a 50% background-corrected isocontour volume of interest (VOI) was used. RCSUVmax was calculated based on the maximum voxel value within the VOI. Additionally, EARL 2 compliance was assessed using RCSUVpeak, which represents the mean activity concentration within a 12 mm diameter spherical VOI positioned to maximise uptake.

EARL compliance was considered reached when all spheres in all locations across the FOV were within the limits defined by EARL for RCSUVmean, RCSUVmax and RCSUVpeak. Where multiple reconstructions fit within the constraints, the reconstruction that would provide the highest image quality (i.e., lowest filter and highest matrix size) was chosen.

To assess the clinical impact of the identified EARL 1 and EARL 2 reconstruction parameters, patient datasets were visually assessed. The first dataset was obtained from an 84-year-old, 69 kg male patient injected with 107 MBq of ^18^F-FDG, acquired using a 10 min static acquisition. The second dataset was from an 88-year-old, 32.5 kg female patient injected with 46 MBq of ^18^F-FDG, acquired using a continuous bed motion (CBM) protocol at a table speed of 1.6 mm/s, selected to approximate the image quality of a 10 min static acquisition as per manufacturer recommendations. Both datasets were reconstructed using the EARL 1 and EARL 2 compliant parameters, as well as the default clinical reconstruction protocol for comparison. Visual assessments were conducted to evaluate differences in image contrast, smoothness, and noise across the reconstruction methods and acquisition protocols. These comparisons aimed to assess the visual implications of EARL compliance on clinical image quality and to compare the relative performance of static and CBM acquisition modes under clinically relevant conditions.

To complement the qualitative assessments, a quantitative comparison of patient data was also performed. For each reconstruction mode (static and CBM) and sensitivity setting (HS and UHS), volumes of interest (VOIs) were placed over 3 representative lesions. Mean and maximum standardised uptake values (SUV_mean_ and SUV_max_) were measured for each ROI and compared to the default clinical reconstructions. The percentage differences relative to the clinical reconstruction were calculated to quantify the impact of EARL-compliant reconstructions on lesion uptake and background.

## 3 Results

Reconstruction parameters meeting both EARL 1 and EARL 2 compliance criteria were successfully identified across all tested scenarios, including static and continuous bed motion (CBM) acquisitions in both High Sensitivity (HS) and Ultra-High Sensitivity (UHS) modes (Table 1). The parameters determined for CBM acquisitions were identical to those for static acquisitions within the same mode, indicating that CBM performance can be optimised to match static imaging for EARL compliance under the given acquisition speeds.

**Table 1:** Parameters for different reconstruction modes that meet EARL1 and EARL2 requirements.

	Acquisition mode	Acquisition type	Matrix size	FWHM of Gaussian (mm)
EARL 1	HS	Static	220	7
	HS	CBM	220	7
	UHS	Static	128	7
	UHS	CBM	128	7
EARL 2	HS	Static	440	5
	HS	CBM	440	5
	UHS	Static	220	4
	UHS	CBM	220	4

All reconstruction use OSEM with 4 iterations and 5 subsets with PSF and TOF corrections. All images are attenuation corrected using CT, scatter corrected and have all other standard corrections.

**Figure 1:**
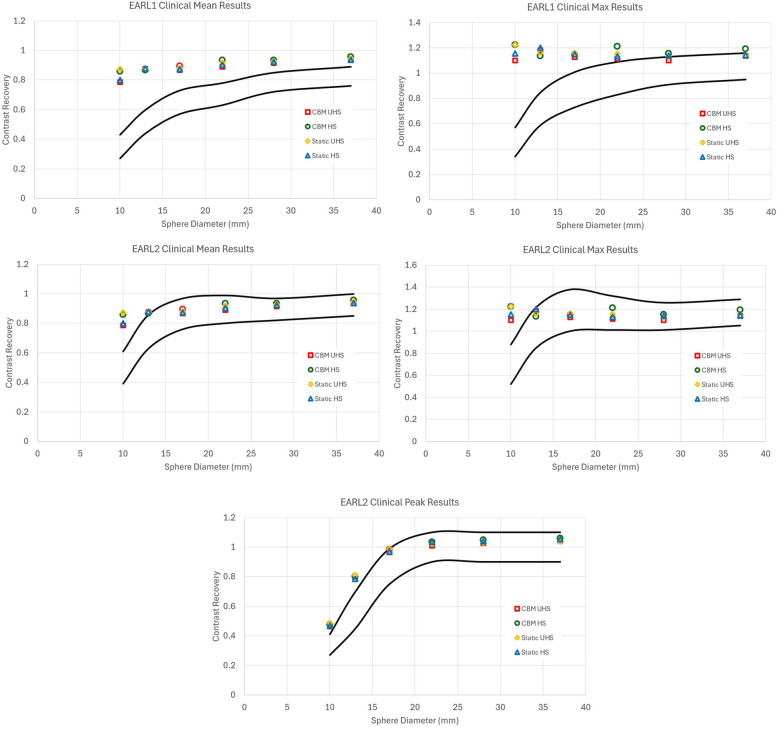
Contrast recovery curves for RCSUVmean, RCSUVmax and for EARL2 only RCSUVpeak using the standard clinical reconstruction parameters for static and CBM in HS and UHS modes. Black lines are the limits set by EARL. All graphs are taken from the centre of the axial FOV. The default clinical reconstructions did not comply with EARL 1 or EARL 2 criteria in any acquisition mode.

**Figure 2:**
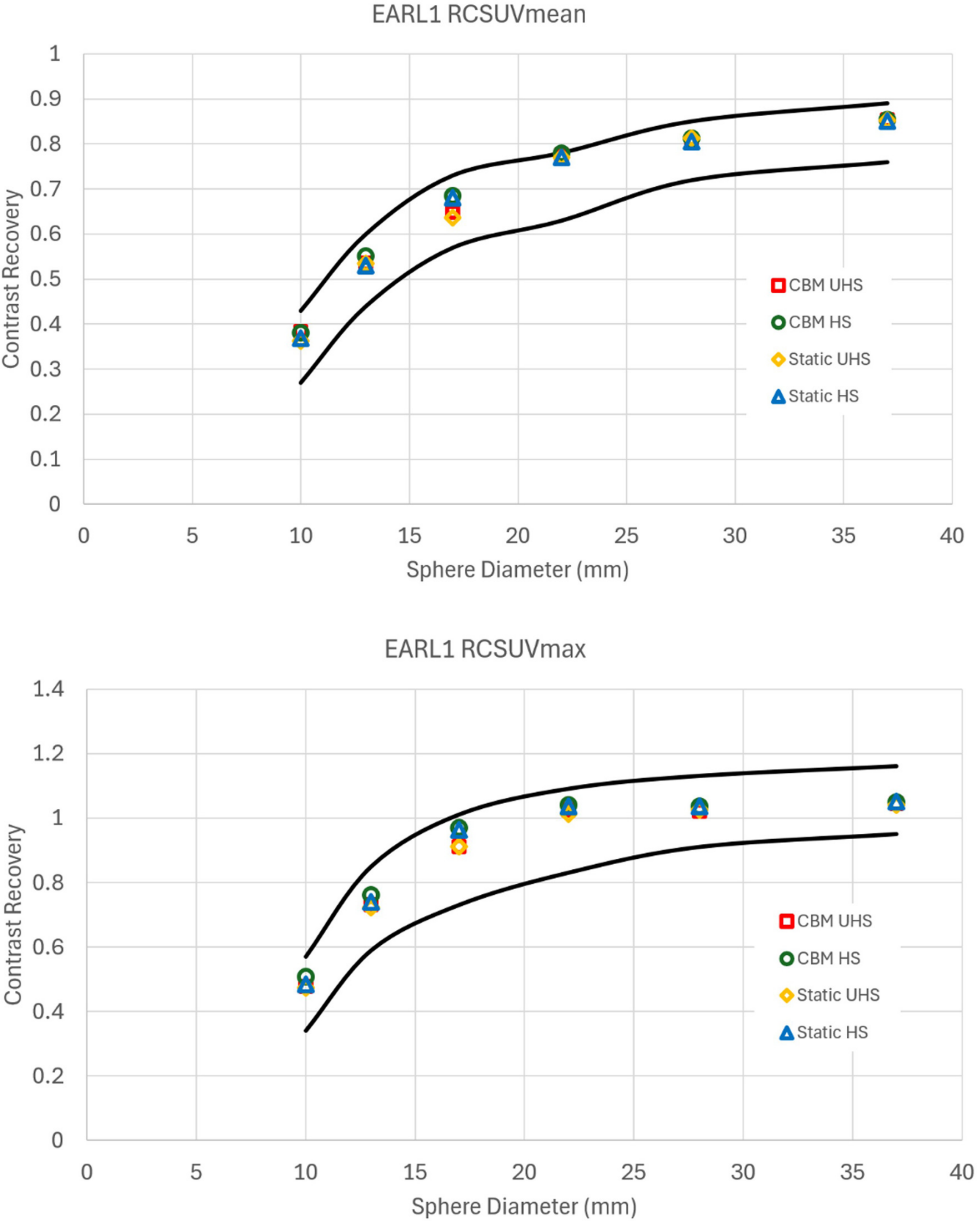
Contrast recovery curves for RCSUVmean and RCSUVmax using the EARL 1 reconstruction parameters for static and CBM in HS and UHS modes. Black lines are the limits set by EARL. All graphs are taken from the centre of the axial FOV.

For EARL 1 compliance, achieving the specified recovery coefficient ranges required significant reductions in image quality compared to the default clinical reconstructions. This reduction was primarily due to the excellent contrast recovery and flatness of the curves on the Quadra (Figure 1). For the EARL 1 criteria, the excellent contrast recovery of the system necessitated a large amount of smoothing and a reduction in matrix size. (Figure 2). The degradation in image quality required was more pronounced in UHS mode compared to HS.

**Figure 3:**
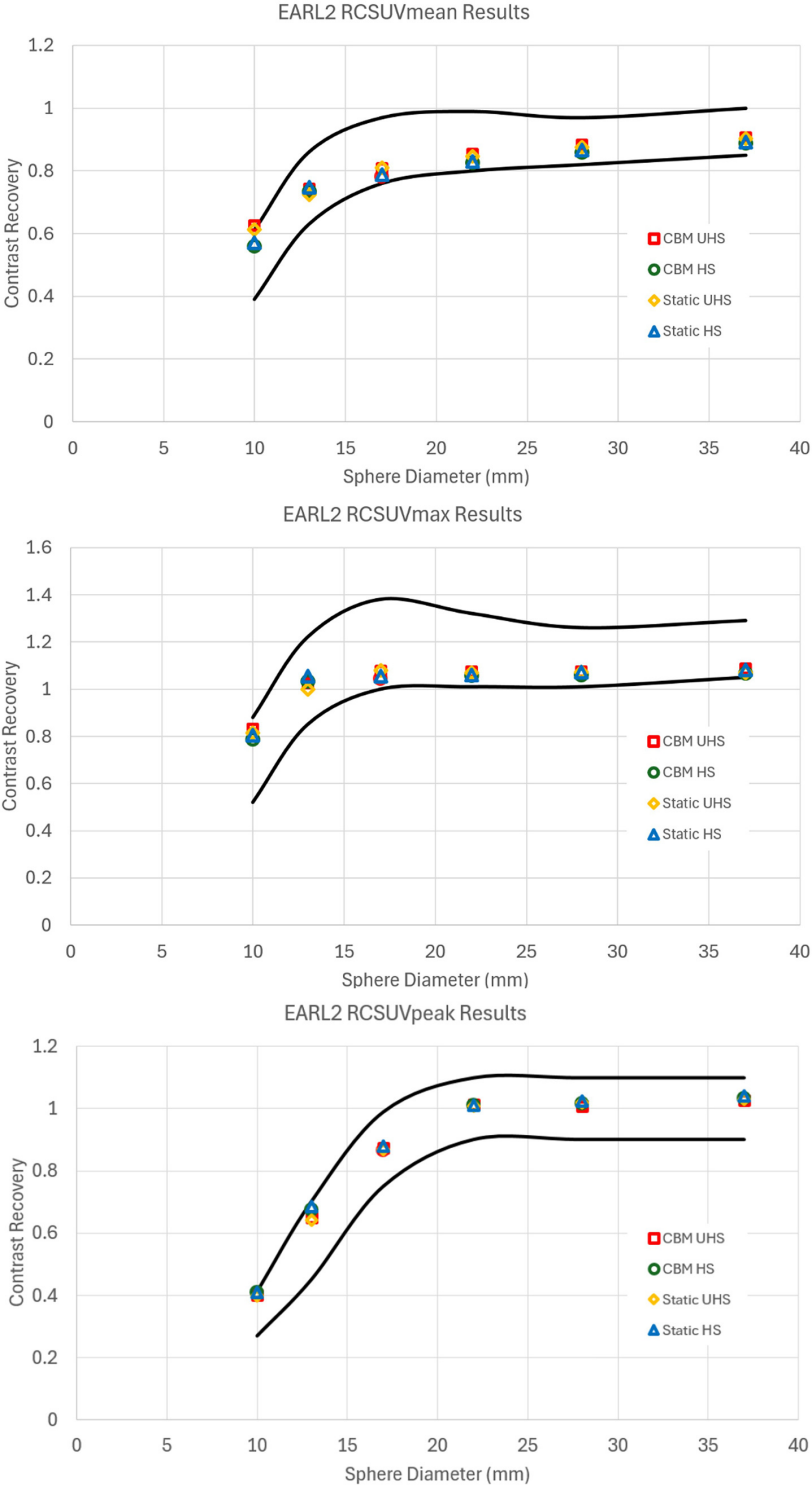
Contrast recovery curves for RCSUVmean, RCSUVmax and RCSUVpeak using the EARL 2 reconstruction parameters for static and CBM in HS and UHS modes. Black lines are the limits set by EARL. All graphs are taken from the centre of the axial FOV.

Similarly, reconstructions meeting EARL 2 compliance also exhibited reductions in image quality relative to clinical reconstructions, though to a lesser extent compared to EARL 1 (Figure 3). The UHS mode required greater smoothing and lower matrix size than HS to meet the recovery coefficient criteria, reinforcing the observation that the increased sensitivity of UHS poses challenges to achieve standardisation under EARL guidelines.

Only one reconstruction parameter set per acquisition type met EARL compliance in most cases. The exceptions were CBM acquisitions in HS and UHS modes for EARL 2, where two parameter sets met the criteria. In these cases, the reconstructions with higher matrix sizes and lower Gaussian filtering were selected to balance compliance with the preservation of spatial resolution and contrast (Table 1).

Contrast recovery coefficients (RCSUVmean) demonstrated variation across the axial FOV, with the highest contrast recovery observed at the centre and progressively lower values toward the edges. The degree of reduction in contrast varied with sphere size. For the largest sphere, the variation in RCSUVmean across the FOV was minimal, ranging from 2% to 5%. In contrast, the smallest sphere exhibited a more pronounced reduction in contrast, with up to a 25% decrease in RCSUVmean in the HS static acquisition.

The static UHS and CBM HS acquisitions exhibited similar patterns of contrast recovery variability across the FOV. However, the UHS CBM acquisition demonstrated a more uniform contrast recovery profile, with a maximum variation of only 11% in RCSUVmean. This improved uniformity aligns with the sensitivity profile of the CBM acquisition in UHS mode, which mitigates some of the axial sensitivity fall-off seen in static and CBM HS acquisitions.

Despite the observed variations, the chosen reconstruction parameters successfully met EARL compliance criteria across the entire axial FOV for all acquisition modes. However, achieving compliance was more challenging in cases where the small sphere exhibited significant variability throughout the axial FOV, necessitating careful parameter optimisation to balance contrast recovery across the FOV while maintaining EARL standards.

**Figure 4:**
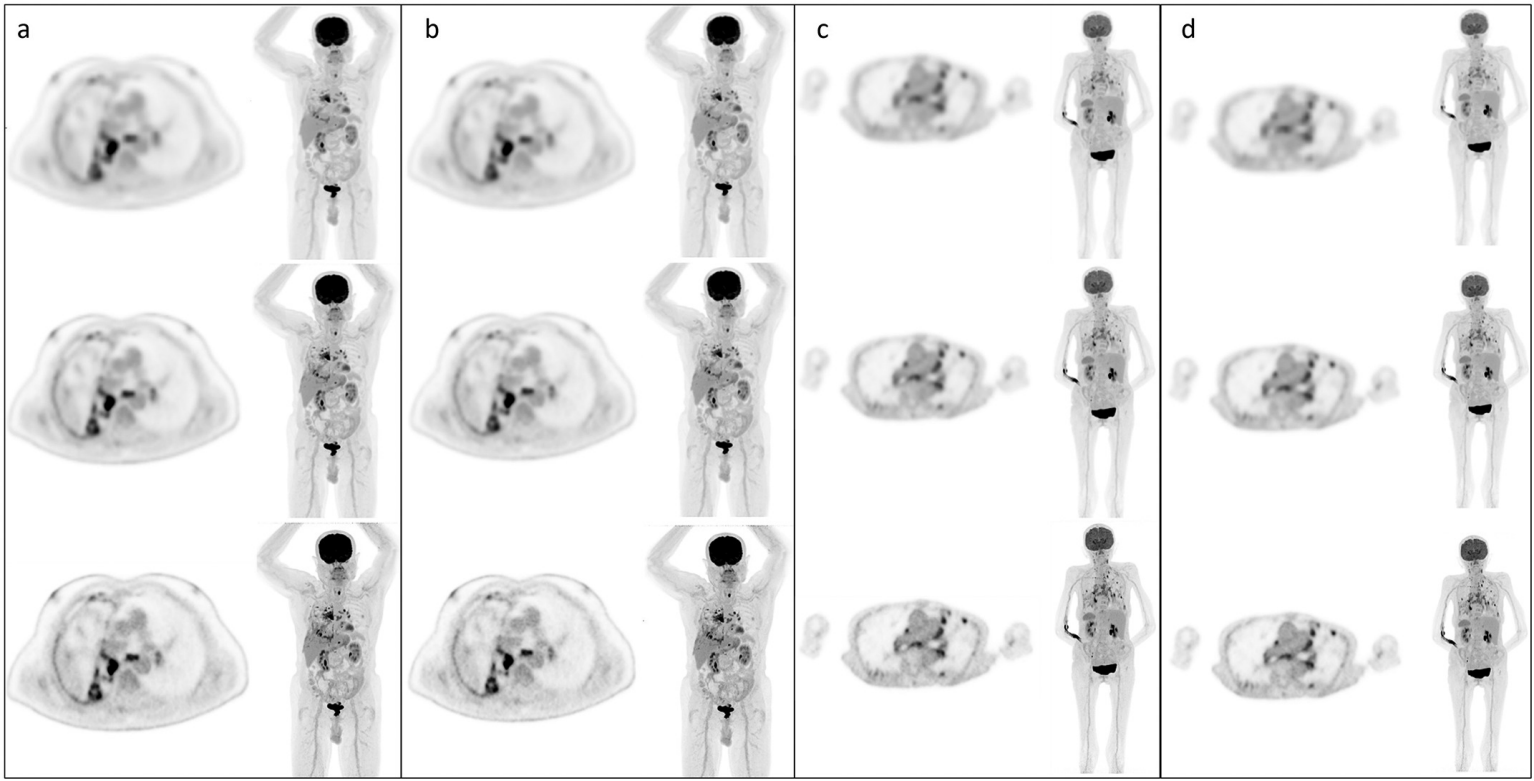
Example static and CBM patient images with each reconstruction type; **(a)** HS images static, **(b)** UHS images static, **(c)** HS images CBM, **(d)** UHS images CBM. In all cases, top row EARL 1 reconstruction parameters, middle row EARL 2 reconstruction parameters, bottom row default clinical reconstruction parameters.

Phantom-based reconstructions that adhered to EARL standards were applied to patient datasets to assess the visual impact of these parameter adjustments (Figures 4). For EARL 1-compliant reconstructions, patient images exhibited noticeable reductions in image quality, with smoother appearance and lower contrast compared to clinical reconstructions. This was particularly apparent in UHS mode, where the degradation was most severe. EARL 2-compliant reconstructions also displayed reductions in image quality, but the visual impact was less pronounced compared to EARL 1.

Quantitative analysis of patient datasets confirmed substantial reductions in measured SUVs for EARL-compliant reconstructions compared to the clinical protocol. For static acquisitions, EARL 1 reconstructions showed average decreases in SUV_mean_ of 31% (UHS) and 39% (HS), and reductions in SUV_max_ of 49% (UHS) and 48% (HS). EARL 2 reconstructions showed less pronounced decreases: SUV_mean_ was reduced by 23% (UHS) and 27% (HS), and SUV_max_ by 26% (UHS) and 32% (HS). For CBM acquisitions, EARL 1 reconstructions demonstrated average SUV_mean_ reductions of 34% (UHS) and 46% (HS), and SUV_max_ reductions of 54% (UHS) and 54% (HS). EARL 2 reconstructions again showed smaller decreases: SUV_mean_ was reduced by 29% (UHS) and 34% (HS), and SUV_max_ by 31% (UHS) and 37% (HS). The variability across VOIs was moderate, with standard deviations ranging between 1% and 7% for all measurements.

## 4 Discussion

In this study, we determined reconstruction parameters to achieve EARL 1 and EARL 2 compliance on the Siemens Biograph Vision Quadra PET-CT using phantom data. Reconstructions were tested across both static and CBM acquisitions in HS and UHS modes. Our analysis demonstrated that EARL compliance could be achieved consistently across all tested scenarios, with identical parameters identified for static and CBM acquisitions within the same mode. Despite these successes, our findings also underscore significant trade-offs between achieving quantitative standardisation and preserving the high-quality imaging capabilities of this advanced system.

One of the most significant challenges highlighted by this study was the need to substantially reduce image quality to meet EARL compliance, particularly for EARL 1. This reduction was especially pronounced in UHS mode, where the system's superior sensitivity and resolution had to be heavily curtailed to fit the originally almost flat contrast recovery curves into the curved shape of the EARL requirements. For EARL 2 compliance, the contrast recovery curve only just fell within the allowable range, with results consistently near the lower boundary of the EARL limits for the larger spheres but near the upper boundary for the smaller spheres. EARL 1 reconstructions required even more smoothing compared to EARL 2, and UHS acquisitions consistently demanded greater smoothing than HS acquisitions to conform to the standards. Nonetheless, when optimised for compliance, the contrast recovery curves across all acquisition modes were similar at the 10:1 sphere-to-background ratio.

These findings highlight the trade-offs between achieving EARL compliance and maintaining optimal image quality. While compliance ensures quantitative standardisation, the resulting reconstructions may not meet the expectations for diagnostic clarity or precision achievable with advanced PET systems. This trade-off is especially critical for high-performance modes like UHS.

The comparison of static and CBM acquisitions revealed no differences in reconstruction parameters required to achieve EARL compliance, reinforcing the versatility of CBM as a viable alternative to static scanning. This finding is particularly relevant for LAFOV systems like the Quadra, where CBM acquisitions may provide a more consistent sensitivity response across the FOV and allow for whole body scans to be performed simply. However, it is worth noting that while EARL standards were met consistently across the FOV, some variation in contrast recovery curves was observed in both HS and UHS, with slightly better performance in the central FOV and worse performance near the edges. Importantly, these variations were not substantial enough to affect compliance results.

The results from this study align with those from two other centres that have performed EARL measurements on the Quadra. The Groningen team (8) evaluated static acquisitions in HS mode, reporting that a 220 × 220 matrix with a 7 mm Gaussian filter achieved EARL 1 compliance, which matches our findings and provides confidence in the robustness of our results. For EARL 2, they found compliance with a 220 × 220 matrix and 5 mm Gaussian filter but did not evaluate a 440 × 440 matrix. Similar to our findings, their EARL 2 results were near the lower boundary of the allowable range due to the difficulty in shaping the flat contrast recovery curves to meet EARL criteria.

The Bern group (7) also evaluated static acquisitions in HS mode but employed a different approach by altering the number of iterations and subsets, which we did not explore in this study. A primary reason for not varying these parameters was to maintain consistency with routine clinical protocols and to focus on evaluating the effects of matrix size and smoothing, which are more directly applicable to standard imaging workflows. While we maintained fixed OSEM parameters for these reasons, we acknowledge that varying iterations and subsets could influence EARL compliance by affecting image noise, convergence, and quantitative accuracy. Exploring this flexibility in future studies may offer opportunities to further optimise image quality while still adhering to standardised protocols. The Bern group achieved EARL compliance using a 440 × 440 matrix with Gaussian filtering but also noted the difficulty in meeting EARL 2 requirements, further emphasising the challenge of adapting advanced systems to these standards.

Although a full quantitative analysis of clinical data would have further enriched this investigation, the use of phantom data alone allows for a rigorous, controlled assessment of reconstruction parameters. Phantom studies enable precise measurement of quantitative accuracy, image uniformity, and noise characteristics, providing a critical foundation for clinical applications. Importantly, the findings from this work can guide future efforts to incorporate clinical validation, ensuring seamless translation into patient studies.

Our visual review of patient data revealed that image quality was consistent across acquisition modes but showed noticeable degradation when compared to the system's full capabilities, particularly in reconstructions meeting EARL 1 compliance. Quantitative measurements of SUV_mean_ and SUV_max_ in patient datasets confirmed that EARL-compliant reconstructions systematically reduced uptake values. Compared to the clinical reconstruction, EARL 1 reconstructions resulted in decreases of up to 54% in SUV_max_ and up to 46% in SUV_mean_, with EARL 2 reconstructions showing reductions of up to 37% and 34%, respectively. The variability across regions of interest was modest, indicating that these effects were consistent and reproducible. These results highlight the substantial impact that EARL standardisation has on quantitative metrics in clinical images, reinforcing the importance of balancing harmonisation with preservation of diagnostic information.

One area not assessed in this work is test–retest of the phantom data to determine the stability of results. This will be the subject of further investigation. However, repetition of the measurements due to the annual requirements of the EARL accreditation scheme has thus far demonstrated consistent results, suggesting no indication of instability.

Overall, this study demonstrates that EARL compliance can be achieved across a range of acquisition and reconstruction settings on the Quadra. However, the adjustments required to meet these standards emphasise the need for careful consideration when standardising imaging protocols, particularly when applying them to patient datasets. Future studies incorporating clinical validation are warranted to further investigate the impact of these findings on diagnostic accuracy and quantitative precision in real-world applications.

The results also emphasise the need for updated standards tailored to the capabilities of modern LAFOV PET-CT systems. EARL 1 and EARL 2 were developed for systems with shorter axial FOVs and lower sensitivity, and their application to state-of-the-art systems like the Quadra imposes limitations that undermine the advantages of advanced sensitivity and resolution. Developing an “EARL 3” standard with flat acceptance ranges for RCSUVmean (0.8–1.0) and RCSUVmax (1.1–1.3), along with a 30% increase in the boundaries of the two smallest spheres for RCSUVpeak, could ensure both quantitative consistency and the preservation of high-quality imaging. This approach would fully leverage the capabilities of LAFOV PET-CT systems for both clinical and research applications.

This work serves as a critical step in understanding the implications of EARL compliance on modern PET-CT systems and highlights the importance of balancing standardisation with diagnostic and research-driven imaging needs.

This study demonstrates that EARL compliance can be achieved on the Biograph Vision Quadra PET-CT across all acquisition modes, but at the cost of significant reductions in image quality, particularly in UHS mode and under EARL 1 standards. These findings highlight the limitations of existing EARL criteria for modern LAFOV systems and emphasise the need for an updated “EARL 3” standard that balances quantitative consistency with the advanced imaging capabilities of next-generation PET-CT systems.

## Data Availability

The original contributions presented in the study are included in the article/Supplementary Material, further inquiries can be directed to the corresponding author.
